# A challenging case of carbapenem resistant *Klebsiella pneumoniae-*related pyogenic liver abscess with capsular polysaccharide hyperproduction: a case report

**DOI:** 10.1186/s12879-024-09314-z

**Published:** 2024-04-23

**Authors:** Maryam Sohrabi, Neda Pirbonyeh, Mahvash Alizade Naini, Alireza Rasekhi, Abbas Ayoub, Zahra Hashemizadeh, Fereshteh Shahcheraghi

**Affiliations:** 1 Department of Bacteriology, Pasteur Institute of Iran, Tehran, Iran; 2 Department of Microbiology, Burn and Wound Healing Research Center, Shiraz University of Medical Sciences, Shiraz, Iran; 3 Department of Bacteriology and Virology, School of Medicine, Shiraz University of Medical Sciences, Shiraz, Iran; 4 Department of Internal Medicine, School of Medicine, Shiraz University of Medical Sciences, Shiraz, Iran; 5 Department of Radiology, Shiraz University of Medical Sciences, Shiraz, Iran

**Keywords:** Carbapenem-resistant, *Klebsiella pneumoniae*, Colistin, Pathogenesis, Capsular polysaccharide

## Abstract

**Background:**

Carbapenem-resistant *Klebsiella pneumoniae* (CRKP) infections are a major public health problem, necessitating the administration of polymyxin E (colistin) as a last-line antibiotic. Meanwhile, the mortality rate associated with colistin-resistant *K. pneumoniae* infections is seriously increasing. On the other hand, importance of administration of carbapenems in promoting colistin resistance in *K. pneumoniae* is unknown.

**Case presentation:**

We report a case of *K. pneumoniae*-related pyogenic liver abscess in which susceptible *K. pneumoniae* transformed into carbapenem- and colistin-resistant *K. pneumoniae* during treatment with imipenem. The case of pyogenic liver abscess was a 50-year-old man with diabetes and liver transplant who was admitted to Abu Ali Sina Hospital in Shiraz. The *K. pneumoniae* isolate responsible for community-acquired pyogenic liver abscess was isolated and identified. The *K. pneumoniae* isolate was sensitive to all tested antibiotics except ampicillin in the antimicrobial susceptibility test and was identified as a non-K1/K2 classical *K. pneumoniae* (cKp) strain. Multilocus sequence typing (MLST) identified the isolate as sequence type 54 (ST54). Based on the patient’s request, he was discharged to continue treatment at another center. After two months, he was readmitted due to fever and progressive constitutional symptoms. During treatment with imipenem, the strain acquired *bla*_OXA−48_ and showed resistance to carbapenems and was identified as a multidrug resistant (MDR) strain. The minimum inhibitory concentration (MIC) test for colistin was performed by broth microdilution method and the strain was sensitive to colistin (MIC < 2 µg/mL). Meanwhile, on blood agar, the colonies had a sticky consistency and adhered to the culture medium (sticky mucoviscous colonies). Quantitative real-time PCR and biofilm formation assay revealed that the CRKP strain increased capsule *wzi* gene expression and produced slime in response to imipenem. Finally, *K. pneumoniae*-related pyogenic liver abscess with resistance to a wide range of antibiotics, including the last-line antibiotics colistin and tigecycline, led to sepsis and death.

**Conclusions:**

Based on this information, can we have a theoretical hypothesis that imipenem is a promoter of resistance to carbapenems and colistin in *K. pneumoniae*? This needs more attention.

## Background

Pyogenic liver abscess (PLA) is a rare infectious disease usually caused by bacterial infection of the liver parenchyma in immunocompromised patients [1]. *Klebsiella pneumoniae* and *Escherichia coli* are the most common etiological agents of PLAs in immunocompromised patients [2, 3]. Liver transplantation and diabetes mellitus (DM) are two known risk factors for the development of PLA [1, 4]. *K. pneumoniae* readily colonizes the intestine and can cross the intestinal barrier to enter the liver via the portal vein system and cause PLA in healthy and immunocompromised individuals [1, 2]. Antibiotic resistance in *K. pneumoniae* arises from several mechanisms, including enzymes that directly inactivate antibiotics (e.g., β-lactamases or aminoglycoside-modifying enzymes), activation of efflux pumps that pump out the antibiotic, and mutations in the antibiotic target [5]. The prevalence of multidrug-resistant *K. pneumoniae* strains producing carbapenemase is increasing [6]. OXA-48 is one of the most common carbapenemases and is frequently detected in *K. pneumoniae* and *E. coli* strains [7]. Treatment of infection caused by carbapenem-resistant *K. pneumoniae* (CRKP) isolates is often limited to colistin as a last-line antibiotic [8]. However, the high mortality rate of CRKP infections due to limited treatment options indicate the urgent need for new strategies to treat *K. pneumoniae* infections [8].

Capsular polysaccharide is one of the most important virulence factors of *K. pneumoniae* [9]. Genes involved in capsule production are located on the *cps* operon (capsular polysaccharide synthesis) of the chromosome [9]. The expression of this gene cluster (from *galF* to *ugd*) is driven by three promoters located upstream of *galF*, *wzi*, and *manC* genes, respectively. The conserved *galf*, *cpsACP*, *wzi*, *wza*, *wzb* and *wzc* genes at the 5’ end of the *cps* gene cluster are involved in the transfer and processing of capsular polysaccharide at the bacterial surface [9]. The Rcs (regulator of capsule synthesis) system is a cell envelope stress response system and is present in many *Enterobacterales* species [10]. The Rcs system is activated by outer membrane damage, lipopolysaccharide (LPS) synthesis defects, and peptidoglycan disruption and regulates the expression of genes involved in capsule biosynthesis, motility, biofilm formation, and virulence [10]. In vitro, *E. coli* increases the expression of *cps* genes in the presence of sub-minimum inhibitory concentrations (sub-MICs) of β-lactams, which inhibit the final steps of peptidoglycan synthesis, and colistin, which disrupts the outer membrane [11]. In particular, the administration of antimicrobial agents that increase the levels of extracellular polysaccharides (EPS) can exacerbate biofilm formation in catheters and cause therapeutic challenges [11, 12].

Antibiotic resistance is a complex process that contributes to pathogenesis. An understanding of pathogenesis may facilitate the identification of antibiotic resistance mechanisms and the development of new therapeutic strategies. A study was conducted to investigate the epidemiology of PLAs in Shiraz, Iran from 2020 to 2022. During the study period, the interesting pathogenesis and different mucoviscous phenotype of *K. pneumoniae* isolates from a case with PLA prompted us to investigate capsule *wzi* gene expression, biofilm formation, and the presence of efflux pumps. We analyzed clinical, radiological, microbiological characteristics, and patient outcome in detail.

## Case presentation

In December 2021, a 50-year-old man with clinical presentation of fever and chills was referred from Tehran and was admitted to the post-transplant ward of Abu Ali Sina Hospital in Shiraz, as the most important referral center for patients with liver diseases in Iran. The patient’s medical history included diabetes mellitus (DM) and liver transplantation due to liver cirrhosis in December 2016. Biochemical tests revealed a white blood cell (WBC) count of 6000/µL, fasting blood sugar (FBS) level of 175 mg/dL, blood urea nitrogen (BUN) of 15 mg/dL, creatinine (Cr) of 1.1 mg/dL, C-reactive protein (CRP) of 96 mg/L, estimated sedimentation rate (ESR) of 105 mm/h, alkaline phosphatase (ALP) of 543 U/L, aspartate transaminase (AST) of 27 U/L, alanine transaminase (ALT) of 33 U/L, direct bilirubin (D. bilirubin) of 0.9 mg/dL, total bilirubin (T. bilirubin) of 2.1 mg/dL, and hemoglobin (Hb) of 8.2 g/dL (Table 1). Computed tomography (CT) revealed a 70 × 70 × 70 mm abscess in the right lobe of the liver. The patient’s critical condition due to diabetes mellitus, and liver transplantation with multiple immunosuppressive drugs, necessitated empirical antibiotic treatment, pending culture and antimicrobial susceptibility report. Therefore, imipenem was prescribed. Treatment was started by administering imipenem-cilastatin 500 mg intravenously (IV) every 12 h and percutaneous catheter drainage of the liver abscess. We cultured the pus sample on MacConkey agar and blood agar and incubated it under aerobic and anaerobic conditions at 37 °C. *K. pneumoniae* was isolated and identified as the only pathogen by standard biochemical tests [13]. The *K. pneumoniae* isolate was confirmed to be *K. pneumoniae* by polymerase chain reaction (PCR) and DNA sequencing of the 16 S rRNA gene [3]. Antimicrobial susceptibility of *K. pneumoniae* isolate was determined by the Kirby-Bauer disk diffusion method according to Clinical Laboratory Standards Institute (CLSI) guidelines [14]. A panel of 12 antimicrobial agents was used, including ampicillin (AMP), ceftriaxone (CRO), cefotaxime (CTX), ceftazidime (CAZ), cefepime (CPM), aztreonam (ATM), imipenem (IMI), meropenem (MEM), ertapenem (ERP), gentamicin (CN), amikacin (AK), and ciprofloxacin (CIP). The *K. pneumoniae* isolate (mucoviscous colonies) was sensitive to all tested antibiotics except ampicillin [*K. pneumoniae* is intrinsically resistant to ampicillin]. On the 14th day of hospitalization, based on the patient’s request, he was discharged, while the patient was advised to continue the treatment with imipenem 500 mg IV every 12 h and follow-up of the pigtail inserted in the liver abscess is necessary. He was admitted to a hospital in Tehran and received antibiotics for three weeks and was discharged with partial recovery.

**Fig. 1:**
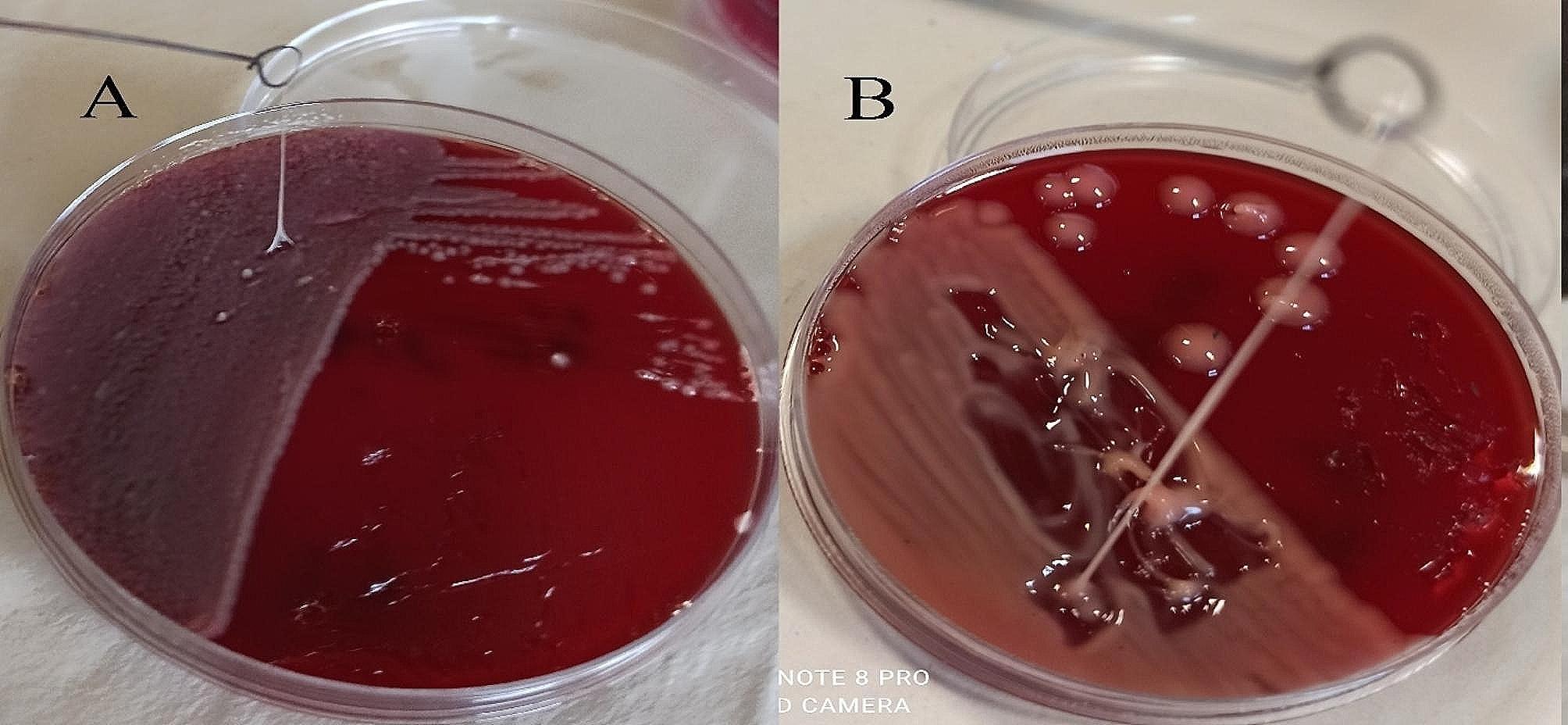
**A.** Sticky mucoviscous colonies of CRKP isolate. **B**. Hypermucoviscous colonies and positive string test of ST23 hvKp strain. A positive string test is defined as the formation of a viscous string > 5 mm in length when colonies grown overnight on a blood agar plate at 37 °C are stretched with a bacteriological loop

After two weeks, the patient had progressive constitutional symptoms and fever. Therefore, he was again referred to Abu Ali Sina Hospital and admitted to the post-transplant ward. In the subdiaphragmatic part of the right lobe of the liver, there was a large liver abscess and pigtail catheter drainage was performed. Biochemical tests also revealed a WBC count of 15,700/µL, BUN level of 40 mg/dL, Cr of 1.8 mg/dL, ALP of 1203 U/L, AST of 84 U/L, ALT of 119 U/L, D. bilirubin of 3.1 mg/dL, T. bilirubin of 5.39 mg/dL, and Hb of 13 g/dL (Table 1). Antibiotic treatment was started by administrating colistin 4.5 × 10^6^ units IV every 12 h and amikacin 500 mg IV every 12 h. Meanwhile, we isolated and identified two species of *K. pneumoniae* and *E. coli* in the pus culture. Here, the phenotypic and genotypic characteristics of the *K. pneumoniae* isolate had changed. On blood agar, the colonies were transparent with a sticky consistency and adhered to the culture medium (sticky mucoviscous colonies) (Fig. 1.A). The isolate was resistant to all tested antibiotics and was identified as a multidrug-resistant (MDR) isolate [15]. The minimum inhibitory concentration (MIC) of imipenem for *K. pneumoniae* was determined to be 128 µg/mL, by broth microdilution method according to CLSI recommendations [14]. MIC test for colistin was also performed by broth microdilution method according to CLSI guidelines [14] and the isolate was sensitive to colistin (MIC < 2 µg/mL). In addition, production of extended-spectrum β-lactamase (ESBL) and carbapenemase was detected by the phenotypic confirmatory disc diffusion test (PCDDT) and the modified carbapenem inactivation method (mCIM) according to CLSI guidelines [14]. Meanwhile, the *E. coli* isolate was also resistant to all tested antibiotics and was identified as an MDR isolate [15]. In *K. pneumoniae* and *E. coli* isolates, virulence genes *entB, ybtS* and antibiotic resistance genes *bla*_TEM_, *bla*_SHV_, *bla*_CTX−M_, and *bla*_OXA−48_ were detected by PCR using the specific primers [3] (Table 2). *K. pneumoniae* isolates belonged to sequence type 54 (ST54) by MLST and were identified as a non-K1/K2 ST54 cKp strain. On the 11th and 18th days of hospitalization, *K. pneumoniae* was again isolated and identified along with *E. coli* in pus culture. On the 20th day, despite the poor condition of the patient, he was discharged with personal consent and insistence and stated that he will continue his hospitalization and treatment in one of the hospitals in Tehran. The patient was advised that it is necessary to continue treatment with colistin 4.5 × 10^6^ units IV every 12 h and amikacin 500 mg IV every 12 h and to follow up the pigtail inserted into the liver abscess.

After 6 weeks, the patient was again referred with pneumocath and shingles and was admitted to the emergency department. The patient stated that he was hospitalized for SARS-CoV-2 infection two weeks ago. The primary diagnosis was pleural effusion, shingles, and liver abscess. On the first day, a CT scan of the chest revealed that all parts of both lung fields were clear without active pulmonary infiltrates or fibrocystic disease. The patient was alert, with stable vital signs, apyretic, vesicular skin lesions in the background of painful erythema (shingles) on the left side of the face and neck, clear lungs, and purulent discharge from the catheter exit site with a positive *K. pneumoniae* culture. The patient’s treatment continued with the administration of colistin, meropenem 500 mg IV every 12 h, and acyclovir. On the sixth day of hospitalization, the patient was alert, apyretic, ulcerative lesions on the face and neck were healing, clear lungs, and a pyogenic liver abscess of 200 × 130 × 61 mm with a positive culture of *K. pneumoniae*. Colistin, imipenem, doxycycline 100 mg every 12 h, and acyclovir were prescribed. Laboratory data revealed a WBC count of 10,200/µL, BUN level of 24 mg/dL, Cr of 1.5 mg/dL, ALP of 1416 U/L, AST of 30 U/L, ALT of 36 U/L, D. bilirubin of 0.6 mg/dL, T. bilirubin of 1 mg/dL, Hb of 7.4 g/dL (Table 1), and subsequently blood transfusion was prescribed to increase blood Hb. On the ninth day, while the liver abscess was very large, surgical drainage was performed. On the 11th day, the patient was alert, with stable vital signs, apyretic, and clear lungs. Purulent secretions were less and *K. pneumoniae* was detected in culture. The patient was receiving colistin, imipenem, tigecycline 50 mg IV every 12 h, fluconazole (used to treat fungal infections) 100 mg every 12 h, and acyclovir. Laboratory data revealed a WBC count of 6400/µL, BUN level of 32 mg/dL, Cr of 1.1 mg/dL, ALP of 1692 U/L, AST of 14 U/L, ALT of 19 U/L, D. bilirubin of 0.7 mg/dL, T. bilirubin of 1.4 mg/dL, Hb of 9.5 g/dL. On the 18th day, the patient was alert, with stable vital signs, apyretic, clear lungs, and shingles lesions were improving. The secretions were brief and *K. pneumoniae* was detected in the culture. Laboratory data revealed a WBC count of 6100/µL, BUN level of 14 mg/dL, Cr of 1.5 mg/dL, ALP of 1628 U/L, AST of 25 U/L, ALT of 45 U/L, D. bilirubin of 0.5 mg/dL, T. bilirubin of 0.9 mg/dL, Hb of 7.3 g/dL, and subsequently blood transfusion was prescribed to increase blood Hb. The patient was receiving colistin, doxycycline, and amikacin. On the 30th day, the patient was alert, with stable vital signs, apyretic, and *K. pneumoniae* was detected in the culture of the abscess secretions and *Enterococcus* sp. in the culture of the wound. On the 42nd day, the patient was alert, with stable vital signs, apyretic, and the shingles lesions were dry and improving. *K. pneumoniae* was detected in culture of liver abscess secretions. The patient was receiving colistin, tigecycline, doxycycline, fluconazole, and linezolid 600 mg every 12 h. On the 47th day, laboratory data revealed a WBC count of 7300/µL, BUN level of 15 mg/dL, Cr of 0.9 mg/dL, CRP of 8 mg/L, ALP of 4353 U/L, AST of 101 U/L, ALT of 208 U/L, D. bilirubin of 6 mg/dL, T. bilirubin of 12 mg/dL, and Hb of 7 g/dL (Table 1). The patient was discharged by personal desire, while all the professors were of the opinion that the patient should remain hospitalized. On the 60th day, the patient died while the clinical findings indicated that PLA was complicated by sepsis.

**Table 1:** Clinical features and laboratory findings of a case of *K. pneumoniae*-related pyogenic liver abscess

Demographic and clinical characteristics					
Age	50 years old				
Sex	Male				
Clinical presentation	Fever and chills				
Computed tomography (CT) findings	A 70 × 70 × 70 mm abscess in the right lobe of the liver				
Underlying diseases	Diabetes mellitus (DM) and liver transplantation				
Initial empirical antibiotic	Imipenem				
Laboratory findings and duration of hospitalization					
Week	In the first week after admission	After eight weeks	After sixteen weeks	After twenty weeks	Reference range
Parameters					
WBC (×10^3^/µL)	6000	15,700	10,200	7300	4.0–10.0
FBS (mg/dL)	175	ND	ND	ND	70–99
BUN (mg/dL)	15	40	24	15	7.0–24.0
Creatinine (mg/dL)	1.1	1.8	1.5	0.9	0.7–1.3
C-reactive protein (mg/L)	96	ND	ND	8	Up to 6
ESR (mm/h)	105	ND	ND	ND	Up to 20
ALP (U/L)	543	1203	1416	4353	40–129
AST (U/L)	27	48	30	101	Up to 40
ALT (U/L)	33	119	36	208	Up to 41
Direct bilirubin (mg/dL)	0.9	3.1	0.6	6	Up to 0.4
Total bilirubin (mg/dL)	2.1	5.39	1	12	< 1.3
Hemoglobin (g/dL)	8.2	13	7.4	7	13.0–17.0

^*^WBC: white blood cell; FBS: fasting blood sugar; BUN: blood urea nitrogen; ESR: estimated sedimentation rate; ALP: alkaline phosphatase; AST: aspartate transaminase; ALT: alanine transaminase; ND: not determined.

**Table 2:** Microbiological features of a case of *K. pneumoniae*-related pyogenic liver abscess

Isolates	Before treatment with imipenem	After treatment with imipenem
	Sensitive cKp isolate (mucoviscous colonies)	MDR cKp isolate (sticky mucoviscous colonies), * E. coli*, and *Enterococcus* sp.
Characteristics of *K. pneumoniae* Antibiotic resistance profile	Ampicillin (AMP)	Ampicillin (AMP), Ceftriaxone (CRO), Cefotaxime (CTX), Ceftazidime (CAZ), Cefepime (CPM), Aztreonam (ATM), Imipenem (IMI), Meropenem (MEM), Ertapenem (ERP), Gentamicin (CN), Amikacin (AK), Ciprofloxacin (CIP)
Antibiotic resistance gene	*bla*_SHV_	*bla*_TEM_, *bla*_SHV_, *bla*_CTX−M_, and *bla*_OXA−48_
Virulence gene	*entB, ybtS*	*entB, ybtS*
Capsular type	Non-K1/K2	Non-K1/K2
Sequence type	ST54	ST54
Biofilm (OD)	Strong (0.77)	Moderate (0.181)
Efflux pump	No efflux pump	No efflux pump
*wzi* gene expression (CT)	29.87	23.12

^*^cKp: classical *K. pneumoniae*; MDR: multidrug-resistant; ST: sequence type; OD: optical density; CT: cycle threshold.

**Fig. 2:**
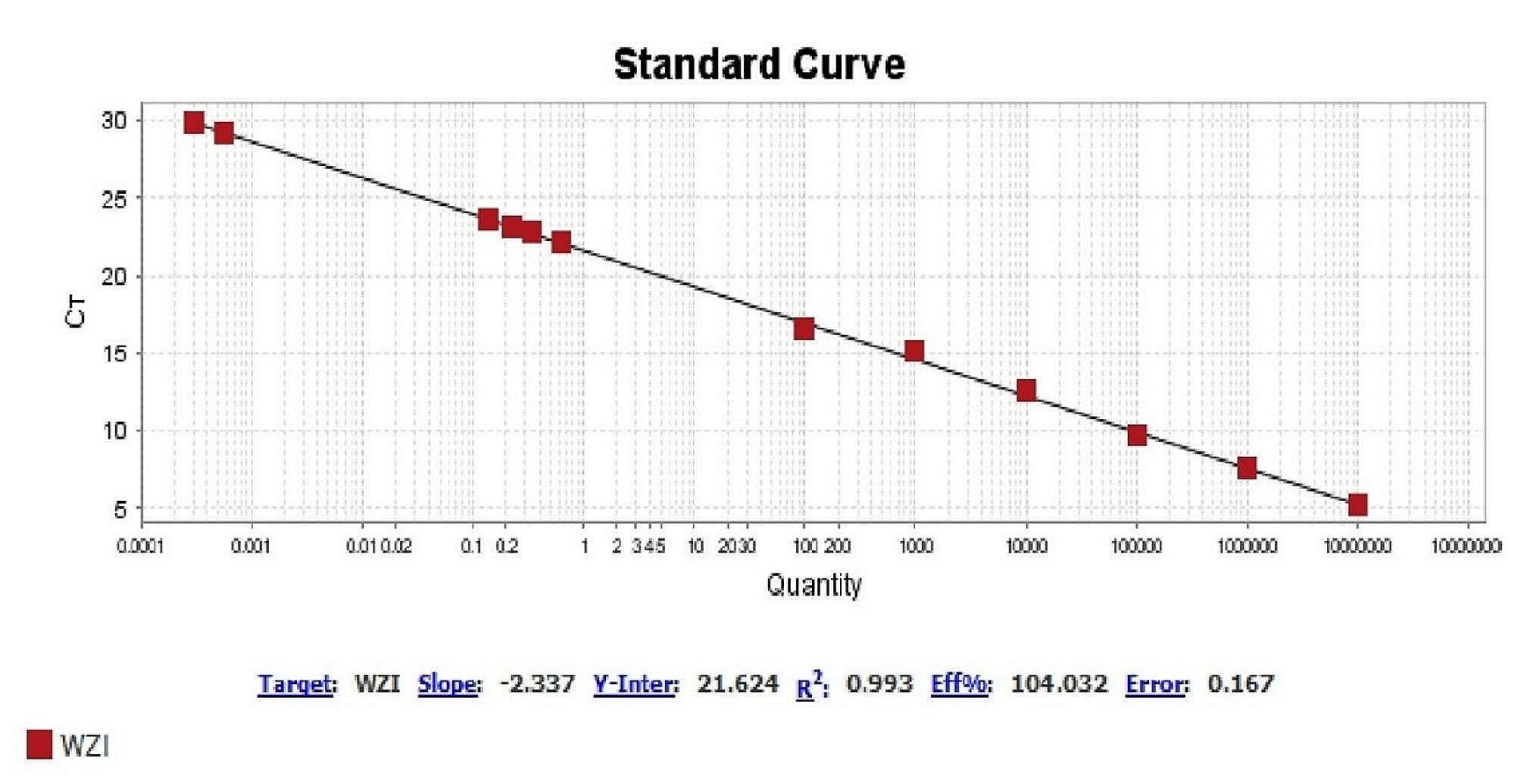
Expression of capsule *wzi* gene in two isolates sensitive (mucoviscous colonies) and CRKP (sticky mucoviscous colonies) and in hvKp strain (hypermucoviscous colonies)

In order to investigate capsular polysaccharide expression, biofilm formation, and the presence of efflux pumps before and after imipenem treatment, a K1 ST23 hypervirulent *K. pneumoniae* (hvKp) strain, previously associated with invasive cryptogenic PLA [3], was included in the present study. HvKp produces a hypercapsule known as the hypermucoviscous phenotype, which is detected by a positive string test [3] (Fig. 1.B). The ability to form biofilm in two isolates sensitive (mucoviscous colonies) and CRKP (sticky mucoviscous colonies) isolated from non-cryptogenic PLA and hvKp isolate (hypermucoviscous colonies) isolated from cryptogenic PLA was investigated according to the previously described microtiter plate method [16]. All three *K. pneumoniae* isolates were biofilm producers. HvKp and sensitive cKp were identified as strong biofilm producers (optical density (OD) was 3.465 and 0.77, respectively), whereas CRKP was identified as a moderate biofilm producer (OD was 0.181) (Table 2). Interestingly, the biofilm formation ability of the hvKp strain was 16-fold higher than that of the positive control strain and almost 5-fold higher than that of sensitive cKp isolate, indicating a higher biofilm formation ability of hvKp compared to cKp. The presence of the efflux pump as an antibiotic resistance mechanism was also investigated according to the previously described ethidium bromide-agar cartwheel method [17]. The efflux pump mechanism could not be detected in all three isolates. Quantitative real-time PCR revealed that the expression of capsule *wzi* gene was nearly the same in hypermucoviscous and sticky mucoviscous isolates (cycle threshold (CT) was 22.47 and 23.12, respectively) and was approximately 1.3-fold that of the mucoviscous isolate (CT was 29.87) (Fig. 2).

## Discussion and conclusions

Classical *K. pneumoniae* (cKp) is associated with non-cryptogenic PLAs in patients with underlying diseases including liver transplantation and diabetes mellitus [2, 3]. Global prevalence of CRKP is increasing, and colistin is considered the last resort antimicrobial to combat CRKP [8]. However, antibiotic resistance is a complex process and there is evidence that antibiotics promote antibiotic resistance [18]. Antibiotics damage bacterial cell structures and induce stress responses such as SOS and Rcs responses [10, 18]. Induction of stress responses leads to emergence of antibiotic resistance [18]. At this time, the role of administration of carbapenems in promoting colistin resistance in *K. pneumoniae* is unknown.

In this study, we report the evolution of a carbapenem-susceptible *K. pneumoniae* strain to CRKP by acquiring *bla*_OXA−48_ during imipenem treatment. Our clinical case highlights the possibility of horizontal gene transfer (HGT) between species during infection. HGT is the major mechanism responsible for the spread of carbapenemase genes through plasmids, transposons, and integrons among bacterial species [19, 20]. As previously reported, several classes of antibiotics (e.g. fluoroquinolones, β-lactams, and aminoglycosides) induce the SOS response at sub-MIC levels. The downstream consequences of induction can lead to the emergence of antibiotic resistance by mutations, biofilm formation, and the development of HGT [18, 21]. This study also showed that imipenem may be a promoter of resistance to carbapenems in *K. pneumoniae*. Unfortunately, due to the limitations of the present study, whole genome sequencing was not performed.

*K. pneumoniae* can acquire resistance to colistin by modifying LPS through addition of cationic groups to lipid A. LPS modification is mainly acquired through chromosomal mutations in the *crrA*/*crrB* or *mgrB* genes [22]. In addition, *K. pneumoniae* increases the amount of capsular polysaccharide and upregulates the transcription of the *cps* operon when grown in vitro in the presence of polymyxin B, leading to increased polymyxin resistance [23]. In vitro studies have shown that exposure to sub-MIC concentrations of β-lactams such as imipenem, which target peptidoglycan, can increase biofilm formation, whereas antibiotics that target the ribosome or DNA replication are less likely to do so [24, 25]. *K. pneumoniae* typically infects patients with indwelling medical devices such as catheters, on which the bacterium can grow as a biofilm [26]. Biofilm development begins with the reversible attachment of planktonic bacteria to a surface, and in the next step, the attachment becomes irreversible and the bacteria multiply and form microcolonies on the surface that begin to produce extracellular polysaccharides (EPS) around the microcolonies [12, 27]. EPS are an insoluble and slimy secretion that are released by bacterial cells and lead to biofilm formation [12]. Bacterial biofilms are a serious problem in medicine because they cause chronic infections due to their high resistance to antibiotics and the host’s defense system [27]. The biofilm structure prevents the penetration of antibiotics. Furthermore, within biofilms, persister cells that are temporarily dormant or grow very slowly are associated with the emergence of antibiotic resistance [27]. According to published research, resistant bacterial cells increase the minimum inhibitory concentration (MIC), whereas no increase in MIC is observed in persister cells [28, 29]. Meanwhile, the ability of invasive hvKp strains to form biofilms is significantly higher than that of non-invasive cKp isolates [30]. In hvKp strains, *rmpA* (regulator of mucoid phenotype) and *rmpA2* genes increase the expression of *cps* genes, resulting in a hypermucoviscous phenotype [31, 32]. An increase in the amount of capsular polysaccharide contributes to the formation of a stronger biofilm in *K. pneumoniae* [30, 33]. But why was the biofilm-forming ability of the CRKP (sticky mucoviscous colonies) isolate, which overexpressed the *cps* operon almost as much as the hvKp strain, reduced to a moderate level? Interestingly, no hypermucoviscous phenotype was also observed in this isolate. Extracellular polysaccharides (EPS) are known as capsule, slime, and glycocalyx [34]. The distinction between capsule and slime is not clear, and glycocalyx is usually used as a general term to refer to EPS [34]. The capsule is tightly bound to the bacterial cell and is not removable by repeated washing, whereas the slime layer is loosely bound to the bacterial cell and is easily washed off [34, 35]. Slime also imparts a sticky consistency to bacterial growth on a solid medium [34, 36]. We observed that CRKP colonies on blood agar had a sticky consistency and adhered to the culture medium. In addition, the capsule was loose and easily washed off during washing, which likely reduced biofilm formation in the biofilm formation assay.

Slime-producing bacterial strains grow embedded in an insoluble glucan matrix associated with surfaces and form very thick biofilms compared to non-slime-producing strains that preferentially grow as non-adherent cells in the culture supernatant [35]. Therefore, slime is involved not only in increasing the initial adhesion, but also in the subsequent formation of microcolonies [35]. The present study shows that imipenem may increase capsular polysaccharide expression and slime production in CRKP strains, which can exacerbate biofilm formation in PLA catheters and promote colistin resistance. It would be of concern if antimicrobial agents used to treat infections exacerbated biofilm formation, thereby protecting bacteria from antimicrobial agents.

Colistin is widely used to treat intra-abdominal infections and sepsis by CRKP [5]. However, the implementation of colistin monotherapy against these infections is associated with the negative outcome of the emergence of colistin-resistant CRKP [5]. Therefore, colistin is usually prescribed in combination therapy protocols with fosfomycin, tigecycline, carbapenems, and aminoglycosides. Combinations of colistin increase their bactericidal effect against CRKP isolates [5, 37]. In addition, cefiderocol, imipenem cilastatin/relebactam, meropenem-vaborbactam, ceftazidime–avibactam, and aztreonam–avibactam are potent alternatives for the treatment of CRKP infections [5]. At the time of the present study, none of these new antibiotics were available in our country.

The limitations of the present study were that the physician’s choice to prescribe certain antibiotics was often unclear, the doubt that the patient followed the treatment in the period after voluntary discharge, and finally, the whole bacterial genome could not be sequenced.

In this case, which was an immunocompromised patient (diabetes mellitus and immunosuppressive drugs), the *K. pneumoniae* infection was not controlled and the patient died. Factors that can be involved in the emergence of antibiotic resistance and the death of this patient: (A) Treatment with inadequate dose of imipenem and emergence of resistance to carbapenems. (B) Low level of immunity and possibility of opportunistic infections. (C) Sudden changes in prescribed antibiotics. (D) The possibility of mixed infection from the beginning. (E) The patient’s involvement in the treatment process due to the problems caused by being away from the place of residence.​.

Based on this information, we have a theoretical hypothesis that imipenem is a promoter of resistance to carbapenems and colistin in *K. pneumoniae*. This needs more attention.

## Data Availability

All relevant data are fully presented in the manuscript.

## Abbreviations

PLA: Pyogenic liver abscess
MDR: Multidrug-resistant
CRKP: Carbapenem-resistant *K. pneumoniae*
EPS: Extracellular polysaccharides
ESBL: Extended-spectrum-β-lactamase
PCDDT: Phenotypic confirmatory disk diffusion test
Mcim: Modified carbapenem inactivation method
ST: Sequence type
HGT: Horizontal gene transfer
